# Palliative care at the end of life: reflections on dignity and peace in light of complexity

**DOI:** 10.1590/0034-7167-2025-0193

**Published:** 2026-06-12

**Authors:** Marilia Alves Furtado, Livia Ponte Correia Mota, Vera Lúcia Mendes de Paula Pessoa, Viviane Magalhães Pereira

**Affiliations:** I Universidade Estadual do Ceará. Fortaleza, Ceará, Brasil; II Universidade de Fortaleza. Fortaleza, Ceará, Brasil

**Keywords:** Palliative Care, End of Life, Dignity, Nursing Theory, Nursing Care., Cuidados Paliativos, Fin de la Vida, Dignidad, Teoría de Enfermería, Atención de Enfermería.

## Abstract

**Objectives:**

reflect on the assumptions of “dignity”, “respect”, and “being at peace” present in the Theory of the Peaceful End of Life, in association with Edgar Morin’s Complex Thought.

**Methods:**

a reflective study, constructed through Edgar Morin’s works, in dialogue with the Theory of the Peaceful End of Life and with pertinent literature on patient care in palliative care.

**Results:**

the following categories were obtained: On complex thought; Dignity and respect: the complex articulation between dependence and autonomy; and Being at peace: the complex paradox intertwined in order and disorder.

**Final Considerations:**

the study allowed consideration of the complexity intertwined in the concepts of dignity and respect at the end of life, and being at peace, understanding that both concepts integrate elements that, at first glance, are contradictory, but complementary.

## RESUMEN

**Objetivos:**

reflexionar sobre los supuestos de “dignidad”, “respeto” y “estar en paz” presentes en la Teoría del Final Pacífico de la Vida, en conjunción con el Pensamiento Complejo de Edgar Morin.

**Métodos:**

estudio reflexivo, construido a partir de la obra de Edgar Morin, en diálogo con la Teoría del Final Pacífico de la Vida y la literatura relevante sobre cuidados paliativos.

**Resultados:**

se obtuvieron las siguientes categorías: Sobre el pensamiento complejo; Dignidad y respeto: la compleja articulación entre dependencia y autonomía; y Estar en paz: la compleja paradoja entrelazada con el orden y el desorden.

**Consideraciones Finales:**

el estudio permitió considerar la complejidad entrelazada de los conceptos de dignidad y respeto al final de la vida, y estar en paz, entendiendo que ambos conceptos integran elementos que, a primera vista, son contradictorios, pero complementarios.

## INTRODUCTION

Palliative care (PC) is an approach aimed at improving the quality of life of individuals facing life-threatening illnesses and/or intense distress related to health issues, including chronic diseases. Not limited to patient care, PC aims to provide family support, as well as physical, psychological, social, and spiritual support. According to the World Health Organization, an estimated 56.8 million people worldwide have PC needs, but only 12% receive it^(1)^.

In order to strengthen PC implementation in healthcare points, greater knowledge and theoretical-philosophical basis regarding such assistance are necessary, leading to reflection by healthcare professionals on their practice and expanding the understanding of palliative principles, as well as the insertion and dissemination of a care practice based on human distress alleviation.

In this context, the use of theories presents itself as a valuable tool for guiding our perspective on a given phenomenon, enabling a comprehensive and reflective process. Considering the context of PC, the Theory of the Peaceful End of Life (TPEL), created in 1998 by nurses Cornelia Ruland and Shirley Moore, stands out. The authors based their approach on the understanding that caring for terminally ill patients involves a careful approach that goes beyond observing individuals’ physical needs. Professionals must adopt a stance of empathy and sensitivity to symptoms of other natures. Based on this process, the theorists listed five prerequisites for achieving a peaceful end of life: absence of pain; experience of comfort; dignity and respect; being at peace; and proximity to important people^(2)^.

Despite the importance of this theoretical framework for the care of patients at the end of life, preliminary searches in databases on the topic demonstrated limited use of this theory, both nationally and internationally, requiring greater depth in the concepts embedded in its assumptions, which would enable a greater understanding and applicability of this theory in different settings and populations.

A close look at the assumptions of the theory in question leads us to reflect objectively on the conceptual components involved in “dignity and respect”, as well as “being at peace”, based on the understanding that these, because they possess a high level of abstraction, deserve a close look based on a complex perspective. In the TPEL, we observe the association of the concepts of dignity and respect, which are distinct concepts, around a single assumption, since, for the theorists, they are whole parts of the process of valuing individuals as a unique and worthy human being. “Being at peace”, on the other hand, has been defined as a feeling of calm and harmony, related to the absence of anxieties, fears, and worries^(2)^, even in situations of disorder.

Furthermore, the definitions of the assumptions above demonstrate that these concepts can be considered as constitutive pillars for consolidating the others, since providing pain-free care, close to important people, and providing comfort can be understood as a consequence of a care process that values the human being in their entirety and aims to promote peace at the end of life. These arguments, therefore, justify the choice of the two assumptions in question.

However, despite the definitions offered by theorists regarding these concepts, it is observed that they sometimes assume a simplistic and reductionist character in relation to them, requiring greater reflection and an attitude of openness to their complexity. Thus, understanding that the process of caring for patients with advanced disease requires an approach that avoids simplifications and reductionisms, or rather, that understands the human being in all their singularity and multifactoriality, the intention is to establish a dialogue with French philosopher Edgar Morin’s thought^(3)^.

This choice is justified because the author addresses, in general, the need for complex thought, and considers, within this paradigm, in particular, autonomy, the foundation of dignity, and the importance of living and dealing with disorder, the basis for being at peace. Furthermore, for Morin, it becomes necessary for the human sciences to accept the challenge of complexity, as there is no simplicity when dealing with issues of a social and human nature, only the simplified^(4)^. In any case, when dealing with extreme situations in life, such as proximity of death, this challenge becomes even more pressing.

## OBJECTIVES

To reflect on the assumptions of “dignity”, “respect”, and “being at peace” present in the TPEL, in association with Edgar Morin’s Complex Thought.

## METHODS

This is a theoretical-reflective study, based on the analysis of the works “*Introduction à la pensée complexe*”^(3)^ and “*Science avec conscience*”^(4)^ by philosopher, anthropologist and sociologist Edgar Morin, in dialogue with the assumptions of “dignity and respect” and “being at peace” present in the TPEL^(2)^ and with pertinent literature on patient care in PC.

To this end, the discussion is structured into three categories, which are: On complex thought; Dignity and respect: the complex articulation between dependence and autonomy; and Being at peace: the complex paradox intertwined in order and disorder.

## DEVELOPMENT

### On complex thought

French philosopher Edgar Morin, in his work “*Introduction à la pensée complexe*”^(3)^, presents what he calls complexity. For Morin, complexity is that which cannot be reduced to a single law or a simple idea, as it is constantly changing and contains contradictions. Therefore, taking on the challenge of complexity means accepting a way of thinking about the world in all its multiple dimensions, without fragmenting truths that are antagonistic yet complementary^(3)^. Thus, complexity goes beyond understanding what is difficult or complicated. Furthermore, it is a form of criticism of Cartesian thought, widely disseminated in scientific work, according to which 1) clarity and distinction, 2) the near elimination of difficulties, 3) the simplification of the starting point, and 4) the aspiration for a complete thought, expressed in the form of laws, are priorities for scientific knowledge^(5)^.

Instead of these rules, Morin lists three principles for understanding complexity: 1) the dialogical principle, in which the notions of order, disorder, and organization highlight that ordered phenomena can arise from turbulence or agitation; 2) the holographic principle, which brings to light the idea that not only does the whole contain the part, but also the part contains the whole, necessitating a circular movement from the parts to the whole and from the whole to the parts; 3) recursive organization, meaning that the effects and products of a phenomenon are necessary for its own existence or cause, i.e., for its self-production and self-organization. This principle introduces the concept that a society is continually produced by interactions among subjects, with interactions feedback onto individuals for their co-production. Thus, the social process constitutes an incessant productive cycle^(3)^.

Given the principles and notions outlined by the philosopher, we see the need to unite complex thought with human and social phenomena, revolutionizing the way we understand and think about individuals. Especially in the healthcare field, and more specifically in the context of end-of-life care, thinking about the complex is a necessity.

The palliative approach continually raises awareness of contradictions and uncertainties, as well as order and disorder. The combination of PC and curative care is an example of concepts that, at first glance, seem antagonistic. A disjunctive perspective, therefore, makes it impossible to unite these two lines of care. However, complexity, as a fabric of heterogeneous parts inseparably associated^(3)^, enables the association between supportive treatment, PC, and curative care, based on the understanding that humankind cannot be reduced to its biological aspect, but that healthcare must be capable of addressing individuals’ multiplicity of needs.

### Dignity and respect: the complex articulation between dependence and autonomy

According to the TPEL, this premise means respecting and valuing individuals as a human being. In the context of terminally ill patient care, this notion involves not subjecting patients to anything that violates their integrity and values^(2)^. However, taking respectful care requires recognizing the otherness of the subject being cared for so that their subjectivity and complexity are considered.

On the other hand, human dignity has a primarily philosophical meaning, understood as an axiological concept, i.e., linked to values and what is good. Furthermore, it is considered the moral foundation for human rights and fundamental rights^(6)^. It is worth noting that dignity is sometimes understood as synonymous with autonomy, although autonomy constitutes a basis for dignity, since the awareness of one’s own worth, an inestimable value, is only possible because individuals have the capacity for self-determination. In other words, autonomy is a constitutive element and the ethical foundation of human dignity^(7)^.

However, a deeper look at the notion of autonomy and human dignity reveals a problem of complexity. Complex thought posits that dependence itself fuels autonomy, such as dependence on education, language, culture, and society, as well as care, especially in end-of-life situations. Thus, being a subject is possessing autonomy, while also being dependent^(3)^. There is clearly a difference between dependence without subjection, which fosters autonomy, and dependence with subjection, which eliminates it.

This means, therefore, that contrary to the simplifying view of autonomy free from dependence and dependence without autonomy, we can conclude that the notion of autonomy can only be conceived in associations of opposites and contradictions, revealing the paradigm of complexity^(4)^. Individuals’ will and conduct are intrinsically linked to various aspects of the human condition^(6)^, not just to their ability to act according to rational laws they have formulated for themselves. Therefore, they cannot be dissociated from the context in which individuals find themselves, their motivations, and relationships. Thus, what is required by this concept of dignity is respect for their uniqueness.

Considering the several dependences faced by individuals experiencing serious illness and at the end of their lives, it would be erroneous and simplistic to consider them devoid of dignity or that they do not demand respect due to their impaired capacity for self-determination and the full exercise of their autonomy. A perspective on such situations, based on complexity, enables healthcare capable of articulating dependence, autonomy, dignity, and respect. Thus, a care approach based on ethics is necessary, including the understanding that asymmetries are inseparable in care relationships, but that ethical care can promote dignity and respect despite dependence. Considering and respecting patients’ previous life history, for instance, can be considered an attitude that promotes dignity and respect for dependent individuals.

In addition to the issue of autonomy, a study carried out by Chochinov *et al*.^(8)^ also demonstrates other aspects of the concept of dignity through its application to specific cases. Patients with advanced cancer were asked about the meaning of dignity. As a result, the authors identified three categories: 1) Disease-related concerns, which relate to the level of independence and the intensity of symptoms experienced, both physical and emotional; 2) Personal resources of dignity, in which patients reported what could be done to maintain dignity, such as professionals’ resilience, hope, autonomy, as well as practices to help them cope with physical and emotional challenges; and 3) Social resources of dignity. This last category addresses relationship issues that enhance the sense of dignity, such as privacy, social support, not feeling like a burden to others, and caring practices in interactions with healthcare professionals.

The definitions listed above by study participants, in addition to confirming the relevant role of autonomy within the feeling of dignity, bring to light the multidimensional and complex nature of the concept, demonstrated by the multiplicity of definitions assumed by subjects, which must be understood in a process of articulation.

The understanding that we are simultaneously physical, biological, social, cultural, psychic, and spiritual beings, through complex thought, makes clear the need to conceive of the articulation, identity, and difference among all these aspects, enabling the effective respect for the beings that present themselves in all the complexity inherent to their human condition. Thus, conceiving dignity and respect at the end of life presupposes an attitude of recognition that such concepts are constructed within the social sphere, where both individuals are in the whole (society) and the whole is in the part (individual), revealing the holographic principle. For the philosopher, life is itself a bundle of qualities that emerge from the interactions and organization between the parts and the whole^(4)^. The whole affects the part, and social interactions can contribute to increasing or decreasing patients’ sense of dignity and respect.

Thus, nursing care based on maintaining dignity and respect for individuals experiencing serious illness and the end of life, as recommended by the TPEL, must consider the various aspects inherent to the concepts of dignity and respect, from a complex perspective. It is necessary to understand that dignity and, consequently, respect for singularities are socially constructed, since individuals are formed through interactions within society. Furthermore, understanding individuals as a complex and singular system, as well as dignified, albeit dependent on external factors, means assuming autonomy in the face of their existential dependence on everything necessary for their autonomy^(4)^, Understanding dependence on care not as a lack of autonomy and dignity, but rather as an essential factor in maintaining individuals’ dignity, especially in extreme situations such as the end of life, in a complex perspective that does not simplify antagonisms. It is, therefore, a question of adopting a complex and ethical view of care, recognizing the singular nature of the individual being cared for-even if extremely dependent-who refuses to be reduced to nurses’ will.

### Being at peace: the complex paradox intertwined in order and disorder

Like dignity and respect, the concept of being at peace presents itself as a premise full of meaning and complex webs. When considering this concept, one might imagine that being at peace is related to everything that implies repetition, constancy, and invariance. This would be the case, following Morin’s thinking, if the universe were pure order. However, it must be understood that not only individuals, but the entire universe, balance between order and disorder, and cannot eliminate chance, uncertainty, or unpredictability. In this regard, according to the author, degeneration is a normal phenomenon, such that the only way to deal with it is to regenerate and reorganize, as a way to combat the processes of disintegration^(3)^. This attitude towards unforeseen events, of self-eco-organization, as he calls it, as in the case of a terminal illness, would be the search for balance in the face of challenges, leading to the reestablishment of internal order and a sense of peace.

Thus, despite the disorder experienced by individuals at the end of life, the feeling of peace can arise in various situations: during the process of self-organization amidst chaos, such as in a clear decision about the course of treatment; in pain control; in conflict resolutions with family and loved ones; in the relationship with God or another religious entity; and in reflection on the meaning of the illness and life itself. Thus, the resolution of medical, psychosocial, and/or spiritual issues often precedes the state of peace^(9)^, demonstrating the articulation between the physical, mental, and spiritual worlds.

Thus, to understand the phenomenon of peace at the end of life, it is necessary to take into consideration the following contradictions: disorder at the biological level, as a subject in the process of dying; and self-generated order, as a subject reorganizing itself amidst uncertainty and chance, redefining roles and relationships, based on an understanding of the principles of recursive organization and order/disorder. Considering peace, therefore, is to assume that individuals will be in conflict with various situations, but will, in such moments, achieve new ways of overcoming them, with new elements.

Thus, care aimed at achieving peace must be anchored in the understanding that contradictions and uncertainties are inherent to the human phenomenon, but that it is within this fabric that one can: fertilize the organization, with care itself, fostering friendship and affection; seek transcendence and living, reliving in a community; and seek emotion as an existential dimension, here understood as the outcome of care and existence. Therefore, we have a culture of peace, which is not passive, but rather a dimension to be lived and cultivated^(10)^.

Thus, despite the definition proposed by the TPEL authors, in which being at peace is understood as the absence of anxieties and stressors, complex thought leads to the understanding of peace as a dynamic and singular movement, since individuals are always coexisting with conflicting and contradictory situations. Therefore, even in the midst of stressful situations, such as serious illness, embracing the complex can lead to care aimed at rearranging individuals amid the disorder generated by the approach of death, through consideration of the various intertwined elements in achieving peace. According to Morin^(4)^, the most complex systems are structures that embrace randomness, disorder, and events, becoming more sensitive to them.

### Study limitations

Since this is a theoretical reflection, this study is limited by the need for empirical evidence regarding its postulations. Research is suggested to assess the sense of dignity and respect among end-of-life patients, based on Morin’s complex perspective, both from the perspective of the person receiving care and the perspective of the multidisciplinary team. Furthermore, studies are suggested to assess the sense of peace and calm among patients in the process of finiteness, even in the presence of stressors inherent to the illness.

### Contributions to nursing, health or public policy

The study facilitates the reflective process of nursing professionals who encounter end-of-life patients in their work processes, enabling a critical look at the dignity, respect, and sense of peace of the being cared for, with the potential to directly impact nursing care for critically ill patients, as reflective thinking underpins practice. Furthermore, it encourages the adoption of valuable theoretical frameworks for end-of-life nursing care, such as TPEL and Complex Thought, encouraging discussion around relevant topics in the field and fostering a critical, non-reductionist perspective.

## FINAL CONSIDERATIONS

The reflection on dignity and respect, and being at peace, demonstrates that such assumptions can be considered as pillars for consolidating a peaceful end of life, since these concepts require attitudes that recognize subjects as worthy of pain-free, comfortable care that preserves their relationships and significant roles, favoring reorganization in the face of illness.

Reflecting on dignity and respect requires, first and foremost, recognizing subjects as integrated beings-mind, body, and spirit-socially constructed. Furthermore, it is necessary to consider the contradictions and complementarity between human autonomy and dependence, understanding that dependences, such as those of care, enable, rather than exclude, the maintenance of human dignity.

Peace, in turn, can be achieved even amid chaos and stressors, through care that enables individuals to reorganize amid the uncertainties raised by illness. In this context, peace at the end of life is understood as a dynamic and singular movement, built within a complex fabric, in which order and disorder take a circular and recursive configuration.

## Data Availability

Not applicable.
