# Supplementary material for: Palliative care at the end of life: reflections on dignity and peace in light of complexity
Source: Rev Bras Enferm. 2026 Jun 12;79:e20250193. doi: 10.1590/0034-7167-2025-0193 (PMC13262874; doi:10.1590/0034-7167-2025-0193)
Supplement: Translation

# Translation: Cuidados paliativos no fim de vida: reflexões sobre dignidade e paz à luz da complexidade

**DOI:** 10.1590/0034-7167-2025-0193pt

**Authors:** Marilia Alves Furtado, Livia Ponte Correia Mota, Vera Lúcia Mendes de Paula Pessoa, Viviane Magalhães Pereira

**Affiliations:** I Universidade Estadual do Ceará. Fortaleza, Ceará, Brasil; II Universidade de Fortaleza. Fortaleza, Ceará, Brasil

**Keywords:** Cuidados Paliativos, Fim de Vida, Dignidade, Teoria de Enfermagem, Cuidados de Enfermagem.

## Abstract

**Objetivos:**

refletir sobre os pressupostos de “dignidade”, “respeito” e “estar em paz” presentes na Teoria do Final de Vida Pacífico, em associação com o Pensamento Complexo de Edgar Morin.

**Métodos:**

estudo reflexivo, construído por meio de obras de Edgar Morin, em diálogo com a Teoria do Final de Vida Pacífico e com a literatura pertinente acerca da temática da assistência ao paciente em cuidado paliativo.

**Resultados:**

obtiveram-se as seguintes categorias: Sobre o pensamento complexo; Dignidade e respeito: a articulação complexa entre dependência e autonomia; e Estar em paz: o paradoxo complexo imbricado na ordem e desordem.

**Considerações Finais:**

o estudo permitiu a consideração sobre a complexidade imbricada nos conceitos de dignidade e respeito no final da vida, e estar em paz, entendendo que ambos os conceitos integram elementos, à primeira vista contraditórios, porém complementares.

## INTRODUÇÃO

Cuidados paliativos (CP) são uma abordagem voltada ao aumento da qualidade de vida de indivíduos que estejam enfrentando doenças ameaçadoras à vida e/ou intenso sofrimento relacionado a questões de saúde, incluindo as doenças crônicas. Não limitado ao cuidado com o paciente, a assistência paliativa visa ao apoio familiar, bem como ao suporte físico, psicológico, social e espiritual. Segundo a Organização Mundial da Saúde, estima-se que 56,8 milhões de pessoas ao redor do mundo possuem necessidades paliativas, mas somente 12% chegam a recebê-lo^(1)^.

Para que a implementação dos CP nos pontos de atenção à saúde seja fortalecida, é necessário um maior conhecimento e embasamento teórico-filosófico acerca de tal assistência, levando à reflexão do profissional de saúde sobre sua práxis e ampliando a compreensão a respeito dos princípios paliativistas, bem como a inserção e difusão de uma prática assistencial baseada no alívio do sofrimento humano.

Nesse contexto, o uso de teorias se apresenta como um valoroso instrumento para orientar o olhar sobre um determinado fenômeno, de maneira a possibilitar o processo compreensivo-reflexivo. Considerando o contexto da assistência paliativa, a Teoria do Final de Vida Pacífico (TFVP), criada em 1998 pelas enfermeiras Cornelia Ruland e Shirley Moore, destaca-se de modo relevante. As autoras se basearam no entendimento de que o cuidado do paciente em processo de terminalidade de doença envolve um olhar atento, o qual deve ir além da observação das necessidades físicas do indivíduo, devendo o profissional assumir uma postura de empatia e sensibilidade para sintomas de outra natureza. A partir deste processo, as teóricas elencaram cinco pressupostos para que se atinja um fim de vida pacífico, os quais são: ausência de dor; experiência do conforto; dignidade e respeito; estar em paz; e proximidade com pessoas importantes^(2)^.

Apesar da importância deste referencial teórico para o cuidado de pacientes no fim de vida, as buscas preliminares em bases de dados acerca do tema demonstraram uso limitado da teoria, tanto em nível nacional quanto em internacional, sendo necessário maior aprofundamento em torno dos conceitos imbricados em seus pressupostos, o que possibilitaria um maior entendimento e aplicabilidade da teoria nos diferentes cenários e populações.

Um olhar atentivo sobre os pressupostos da teoria em questão leva-nos ao propósito de refletir objetivamente sobre os componentes conceituais implicados em “dignidade e respeito”, assim como em “estar em paz”, a partir do entendimento de que estes, por possuírem um alto nível de abstração, merecem um olhar atento e embasado em uma perspectiva complexa. Na TFVP, observamos a associação dos conceitos de dignidade e respeito, que são conceitos distintos, em torno de um único pressuposto, visto que, para as teóricas, eles são partes integrantes do processo de valorização do indivíduo como um ser humano singular e digno de valor. O “estar em paz”, por outro lado, foi definido como o sentimento de calmaria e harmonia, relacionando-se à ausência de ansiedades, medos e preocupações^(2)^, inclusive ante a situações de desordem.

Ademais, as definições dos pressupostos acima demonstram que tais conceitos podem ser considerados como pilares constitutivos para consolidação dos demais, uma vez que proporcionar um cuidado livre de dor, próximo às pessoas importantes e com conforto, pode ser entendido como consequência de um processo de cuidado que valoriza o ser humano em toda sua integralidade voltado à promoção da paz no fim de vida. Esses argumentos justificam, portanto, a escolha dos dois pressupostos em questão.

No entanto, apesar das definições elencadas pelas teóricas sobre esses conceitos, observa-se que as mesmas assumem, por vezes, um caráter simplificador e reducionista em relação a eles, sendo necessários um maior aprofundamento reflexivo e uma atitude de abertura à sua complexidade. Dessa forma, entendendo que o processo de cuidado ao paciente com doença avançada requer uma atitude que evite simplificações e reducionismos, ou melhor, que compreenda o ser humano em toda sua singularidade e multifatorialidade, intenciona-se o estabelecimento de um diálogo com o pensamento do filósofo francês Edgar Morin^(3)^.

Essa escolha se justifica porque o autor aborda, em geral, a necessidade do pensamento complexo, e considera, dentro desse paradigma, em particular, a autonomia, fundamento da dignidade, e a importância de viver e lidar com a desordem, base para o estar em paz. Além disso, para Morin, torna-se necessário que as ciências humanas aceitem o desafio da complexidade, pois não há o simples quando se tratam de questões de natureza social e humana, existindo tão somente o simplificado^(4)^. Em todo caso, quando se tratam de situações-limite da vida, como a proximidade da morte, esse desafio se torna ainda mais premente.

## OBJETIVOS

Refletir sobre os pressupostos de “dignidade”, “respeito” e o “estar em paz” presentes na TFVP, em associação com o Pensamento Complexo de Edgar Morin.

## MÉTODOS

Trata-se de estudo teórico-reflexivo, fundamentado na análise das obras “Introdução ao pensamento complexo”^(3)^ e “Ciência com consciência”^(4)^ do filósofo, antropólogo e sociólogo Edgar Morin, em diálogo com os pressupostos de “dignidade e respeito” e o “estar em paz” presentes na TFVP^(2)^ e com a literatura pertinente acerca da temática do cuidado ao paciente em CP.

Para tanto, a discussão se encontra configurada em três categorias, as quais são: Sobre o pensamento complexo; Dignidade e respeito: a articulação complexa entre dependência e autonomia; e Estar em paz: o paradoxo complexo imbricado na ordem e desordem.

## DESENVOLVIMENTO

### Sobre o pensamento complexo

O filósofo francês Edgar Morin, em sua obra “Introdução ao pensamento complexo”^(3)^, apresenta o que ele nomeia de complexidade. Para Morin, o complexo é aquilo que não pode ser reduzido a uma única lei ou a uma simples ideia, pois está em constante transformação e comporta contradições. Portanto, assumir o desafio da complexidade significa admitir uma forma de pensar o mundo em todas as suas múltiplas dimensões, sem fragmentar verdades que são antagônicas, mas, ao mesmo tempo, complementares^(3)^. Assim, a complexidade vai além do entendimento do que é difícil ou complicado. Além disso, trata-se de uma forma de crítica ao pensamento cartesiano, amplamente difundido no trabalho científico, segundo o qual 1) a clareza e distinção, 2) a quase eliminação das dificuldades, 3) a simplificação do ponto de partida e 4) a aspiração de um pensamento completo, expresso na forma de leis, são prioridades para o conhecimento científico^(5)^.

Ao invés dessas regras, Morin elenca três princípios para o entendimento da complexidade, os quais são: 1) princípio dialógico, no qual as noções de ordem, desordem e organização destacam que fenômenos ordenados podem ser originados a partir de uma turbulência ou agitação; 2) princípio hologramático, o qual traz à tona a ideia de que não somente o todo contém a parte, mas também a parte contém o todo, sendo necessário assumir um movimento circular da partes para o todo e do todo para as partes; 3) organização recursiva, significando que os efeitos e produtos de um fenômeno são necessários à sua própria existência ou causa, ou seja, à sua autoprodução e auto-organização. Este princípio introduz o conceito de que uma sociedade é continuamente produzida pelas interações entre os sujeitos, com as interações retroagindo sobre os indivíduos para sua coprodução. Assim, o processo social constitui-se de um círculo produtivo incessante^(3)^.

Diante dos princípios e noções elencadas pelo filósofo, percebe-se a necessidade de unir o pensamento complexo aos fenômenos humanos e sociais, com uma revolução na forma de entender e pensar o sujeito. Especialmente na área da saúde, e mais especificamente no cenário dos cuidados de fim de vida, pensar o complexo é uma necessidade.

A abordagem paliativa suscita continuamente o olhar para contradições e incertezas, além da ordem e da desordem. A junção de assistência paliativa e cuidados curativos é um exemplo de conceitos que, à primeira vista, parecem antagônicos. Um olhar disjuntivo, portanto, torna incapaz de unir estas duas linhas de cuidado. No entanto, a complexidade, como um tecido de partes heterogêneas associadas de forma inseparável^(3)^, possibilita a associação entre tratamento de suporte, CP e cuidado curativo, a partir do entendimento de que o homem não pode ser reduzido ao aspecto biológico, mas que uma assistência em saúde deve ser capaz de assumir a multiplicidade de necessidades do indivíduo.

### Dignidade e respeito: a articulação complexa entre dependência e autonomia

Segundo a TFVP, o presente pressuposto significa o respeito e valorização do indivíduo como um ser humano. No contexto do cuidado ao paciente em terminalidade de doença, tal noção envolve não submeter o paciente a nada que viole sua integridade e valores^(2)^. Assumir um cuidado respeitoso, entretanto, exige o reconhecimento da alteridade do sujeito do qual se cuida, de forma que sua subjetividade e complexidade própria sejam consideradas.

Por outro lado, a dignidade da pessoa humana possui como significado primeiro o filosófico, sendo entendida como um conceito axiológico, ou seja, ligado a valores e àquilo que é bom. Além disso, ela é considerada o alicerce moral para fundamentação dos direitos humanos e dos direitos fundamentais^(6)^. Vale destacar que, por vezes, a dignidade é entendida como sinônimo de autonomia, embora esta se constitua como uma base para a dignidade, já que a consciência do próprio valor, um valor inestimável, só é possível porque o sujeito tem a capacidade de autodeterminação. Em outros termos, a autonomia é um elemento constitutivo e o fundamento ético da dignidade humana^(7)^.

Entretanto, um olhar aprofundado para a noção de autonomia e da dignidade humana denota um problema de complexidade. O pensamento complexo postula que a dependência em si alimenta a autonomia, como a dependência educacional, da linguagem, da cultura e da sociedade, bem como do cuidado, especialmente nas situações de fim de vida. Assim, ser sujeito é possuir autonomia, ao mesmo tempo que ser também dependente^(3)^. Há claramente uma diferença entre a dependência sem sujeição, que alimenta a autonomia, e a dependência com sujeição, que a elimina.

Isso significa, portanto, que contrário à visão simplificadora sobre uma autonomia livre de dependência e uma dependência sem autonomia, podemos concluir que a noção de autonomia só pode ser concebida em associações de opostos e contradições, revelando o paradigma da complexidade^(4)^. A própria vontade e conduta dos indivíduos se encontra em associação intrínseca aos diversos aspectos da condição humana^(6)^, não apenas à sua capacidade de agir segundo leis racionais que formulou para si mesmo, não podendo, portanto, ser dissociada do contexto em que o sujeito se encontra, suas motivações e relacionamentos. Dessa forma, o que se exige com esse conceito de dignidade é o respeito à sua singularidade.

Considerando as múltiplas dependências enfrentadas pelos indivíduos que se encontram em processo de grave adoecimento e no fim de vida, seria errôneo e simplificador considerar que estes estejam destituídos de dignidade ou que não demandam respeito, devido à sua capacidade prejudicada de autodeterminação e de exercer plenamente sua autonomia. Um olhar para tais situações, baseado na complexidade, possibilita uma assistência em saúde capaz de articular dependência, autonomia, dignidade e respeito. Dessa forma, torna-se necessária uma postura de cuidado baseada em uma ética que inclua o entendimento de que assimetrias são indissociáveis em relações de cuidado, mas que uma assistência ética pode promover a dignidade e o respeito, apesar da dependência. Considerar e respeitar a biografia prévia de vida do paciente, por exemplo, pode ser considerada uma atitude para promoção da dignidade e do respeito ao sujeito que se encontra dependente.

Além da questão da autonomia, estudo realizado por Chochinov *et al*.^(8)^ demonstra ainda outros aspectos presentes no conceito de dignidade a partir de sua aplicação a casos concretos. Pacientes com câncer em estágio avançado foram questionados acerca do significado de dignidade. Como resultado, os autores encontraram três categorias: 1) Preocupações relacionadas à doença, a qual diz respeito ao nível de independência e à intensidade de sintomas vivenciados, tanto físicos quanto emocionais; 2) Recursos pessoais de dignidade, na qual os pacientes relataram aquilo que poderia ser realizado para manutenção da dignidade, como resiliência, esperança, autonomia, bem como a prática dos profissionais para auxiliar no enfrentamento dos desafios físicos e emocionais; e 3) Recursos sociais da dignidade. Esta última categoria aborda as questões de relacionamento que aumentam a sensação de dignidade, como privacidade, suporte social, não se sentir um peso para os outros e práticas de cuidado na interação com os profissionais de saúde.

As definições acima elencadas pelos participantes do estudo, além de confirmarem o papel relevante da autonomia dentro da sensação de dignidade, trazem à tona o caráter multidimensional e complexo do conceito, demonstrado pela multiplicidade de definições assumidas pelos sujeitos, as quais devem ser entendidas em um processo de articulação.

O entendimento de que somos, ao mesmo tempo, seres físicos, biológicos, sociais, culturais, psíquicos e espirituais, através do pensamento complexo, torna clara a necessidade de se conceber a articulação, a identidade e a diferença entre todos esses aspectos, tornando possível a efetivação do respeito ao ser que se apresenta em toda a complexidade inerente à sua condição humana. Dessa forma, conceber a dignidade e o respeito no fim de vida pressupõe uma atitude de reconhecimento de que tais conceitos são construídos no âmbito social, onde tanto o indivíduo está no todo (sociedade) quanto o todo está na parte (indivíduo), revelando o princípio hologramático. Para o filósofo, a vida é por si mesma um feixe de qualidades que emerge das interações e da organização entre as partes e o todo^(4)^. O todo retroage sobre a parte, podendo as interações sociais concorrer para o aumento ou a diminuição da sensação de dignidade e respeito do paciente.

Assim, um cuidado de enfermagem embasado na manutenção da dignidade e do respeito ao sujeito em adoecimento grave e fim de vida, conforme preconizado pela TFVP, deve levar em consideração os diversos aspectos inerentes aos conceitos de dignidade e respeito, a partir de uma visão complexa. É necessário o entendimento de que a dignidade e, como consequência, o respeito às singularidades possuem uma construção social, visto que a formação do indivíduo se dá pelas interações que acontecem na sociedade. Além disso, entender o sujeito como um sistema complexo e singular, bem como digno, ainda que dependente de fatores externos, significa assumir sua autonomia diante da sua dependência existencial de tudo aquilo que é necessário à sua autonomia^(4)^, compreendendo a dependência do cuidado não como ausência de autonomia e dignidade, mas sim como um fator essencial para manutenção do sujeito digno, especialmente em situações-limite como o fim de vida, em uma visão complexa e que não simplifica antagonismos. Trata-se, portanto, de assumir uma visão complexa e ética do cuidado, reconhecendo a natureza singular do sujeito do qual se cuida - ainda que em extrema dependência - que se recusa a ser reduzida à vontade do enfermeiro.

### Estar em paz: o paradoxo complexo imbricado na ordem e desordem

Tal qual a dignidade e o respeito, o conceito de estar em paz se apresenta como um pressuposto pleno de significado e teias complexas. Ao se pensar em tal conceito, pode-se imaginar que estar em paz tenha relação com tudo o que implica repetição, constância e invariância. Esse seria o caso, seguindo o pensamento de Morin, se o universo fosse pura ordem. Contudo, há de se entender que, não apenas o sujeito, mas o universo inteiro equilibra-se entre ordem e desordem, não podendo eliminar o acaso, o incerto, a imprevisibilidade. Nesse sentido, segundo o autor, a degeneração é um fenômeno normal, de tal modo que a única maneira de lidar com ela está na atitude de se regenerar e reorganizar, como um combate aos processos de desintegração^(3)^. Tal atitude diante dos imprevistos, de auto-eco-organização, como ele a chama, como no caso de uma doença terminal, seria a busca pelo equilíbrio diante dos desafios, levando ao restabelecimento da ordem interna e à sensação de paz.

Dessa forma, apesar da desordem experimentada pelo indivíduo no fim de vida, o sentimento de paz pôde surgir em diversas situações: durante o processo de auto-organização do sujeito em meio ao caos, como em uma decisão clara sobre o curso do tratamento; no controle da dor; em resoluções de conflitos com familiares e pessoas queridas; na relação com Deus ou outra entidade religiosa; e na reflexão sobre o significado da doença e da própria vida. Assim, a resolução de questões médicas, psicossociais e/ou espirituais costuma anteceder o estado de paz^(9)^, demonstrando a articulação entre os mundos físico, mental e espiritual.

Dessa forma, para o entendimento do fenômeno da paz no fim de vida, é necessário supor as seguintes contradições: a desordem no nível biológico, enquanto sujeito em processo de morte; e a ordem autogerada, como sujeito que se reorganiza em meio às incertezas e acasos, ressignificando papéis e relações, a partir do entendimento dos princípios de organização recursiva e ordem/desordem. Considerar a paz, portanto, é supor que o indivíduo estará em conflito com diversas situações, porém, alcançará, em tais momentos, novas formas de superação, com novos elementos.

Dessa forma, um cuidado que vise ao alcance da paz deverá ser ancorado no entendimento de que as contradições e incertezas são inerentes ao fenômeno humano, mas que é nesta tessitura que se pode: fecundar a organização, com o próprio cuidado, estimulando a amizade e o afeto; buscar pela transcendência e o viver, revivendo em uma comunidade; e buscar pela emoção como dimensão existencial, aqui compreendida como o resultado entre o cuidado e o existir. Portanto, tem-se uma cultura de paz, a qual não é passiva, mas sim uma dimensão a ser vivida e cultivada^(10)^.

Dessa maneira, apesar da definição proposta pelas autoras da TFVP, na qual o estar em paz é entendido como ausência de ansiedades e estressores, o pensamento complexo leva à compreensão da paz como um movimento dinâmico e singular, posto que o sujeito está sempre em coexistência com situações conflituosas e contraditórias próprias. Portanto, ainda que em meio a situações estressoras, como o adoecimento grave, assumir o complexo pode levar a um cuidado que vise à reorganização do indivíduo em meio à desordem gerada pela aproximação da morte, através da consideração dos diversos elementos imbricados no alcance da paz. Segundo Morin^(4)^, os sistemas mais complexos são estruturas que acolhem a aleatoriedade, a desordem e o acontecimento, tornando-se mais sensíveis a eles.

### Limitações do estudo

Por trata-se de reflexão teórica, o presente estudo possui como limitações a necessidade de comprovação empírica acerca do que é postulado, sendo sugerida a realização de pesquisas que avaliem a sensação de dignidade e respeito de pacientes no fim de vida, a partir da visão complexa proposta por Morin, tanto através da perspectiva do ser cuidado quanto da perspectiva da equipe multiprofissional. Além disso, sugere-se a realização de estudos que avaliem a sensação de paz e calmaria de pacientes em processo de finitude da vida, ainda que na presença de estressores inerentes à condição de adoecimento.

### Contribuições para as áreas da enfermagem, saúde ou políticas públicas

O estudo possibilita o processo reflexivo dos profissionais de enfermagem que se deparam com pacientes no fim de vida em seus processos de trabalho, tornando possível um olhar crítico para dignidade, respeito e sensação de paz do ser do qual se cuida, com potencial de impactar diretamente o cuidado de enfermagem de pacientes graves, uma vez que o movimento reflexivo fundamenta a prática. Além disso, estimula a adoção de valorosos referenciais teóricos para o cuidado de enfermagem de pacientes no fim de vida, como a TFVP e o Pensamento Complexo, incitando a discussão em torno de temas relevantes para a área, e estimulando um olhar crítico e não reducionista.

## CONSIDERAÇÕES FINAIS

A reflexão sobre dignidade e respeito, e estar em paz demonstra que tais pressupostos podem ser considerados como pilares para consolidação de um fim de vida pacífico, visto que esses conceitos exigem atitudes que reconheçam o sujeito como digno de um cuidado livre de dor, confortável e que preserve suas relações e papéis significativos, favorecendo a reorganização diante do adoecimento.

Refletir sobre dignidade e respeito exige, antes de tudo, reconhecer o sujeito como um ser integrado - mente, corpo e espírito - construído socialmente. Ademais, é necessário considerar as contradições e a complementaridade entre autonomia humana e dependência, compreendendo que as dependências, como aquelas do cuidado, possibilitam, e não excluem, a manutenção da dignidade da pessoa humana.

A paz, por sua vez, pode ser alcançada ainda que em meio ao caos e na presença de estressores, através de um cuidado que possibilite a reorganização do indivíduo em meio às incertezas suscitadas pelo adoecimento. Nesse ínterim, entende-se a paz no fim de vida como um movimento dinâmico e singular, construída em um tecido complexo, no qual a ordem e desordem assumem uma conformação circular e recursiva.

## Data Availability

Não se aplica.
